# Open AI meets open notes: surveillance capitalism, patient privacy and online record access

**DOI:** 10.1136/jme-2023-109574

**Published:** 2023-11-23

**Authors:** Charlotte Blease

**Affiliations:** 1 Women's and Children's Health, Uppsala Universitet, Uppsala, Sweden; 2 Digital Psychiatry, Beth Israel Deaconess Medical Center, Boston, Massachusetts, USA

**Keywords:** Ethics- Medical, Informed Consent, Information Technology, Education, Policy

## Abstract

Patient online record access (ORA) is spreading worldwide, and in some countries, including Sweden, and the USA, access is advanced with patients obtaining rapid access to their full records. In the UK context, from 31 October 2023 as part of the new NHS England general practitioner (GP) contract it will be mandatory for GPs to offer ORA to patients aged 16 and older. Patients report many benefits from reading their clinical records including feeling more empowered, better understanding and remembering their treatment plan, and greater awareness about medications including possible adverse effects. However, a variety of indirect evidence suggests these benefits are unlikely to accrue without supplementation from internet-based resources. Using such routes to augment interpretation of the data and notes housed in electronic health records, however, comes with trade-offs in terms of exposing sensitive patient information to internet corporations. Furthermore, increased work burdens on clinicians, including the unique demands of ORA, combined with the easy availability and capability of a new generation of large language model (LLM)-powered chatbots, create a perfect collision course for exposing sensitive patient information to private tech companies. This paper surveys how ORA intersects with internet associated privacy risks and offers a variety of multilevel suggestions for how these risks might be better mitigated.

Online record access (ORA) is expanding worldwide. Already patients in an estimated 30 countries can access at least some of their online records via secure portals and apps. Access can include list of medications, vaccinations, laboratory results and even the very narrative reports written by clinicians (the latter commonly referred to as ‘open notes’). In the Nordic countries and the USA, this innovation is advanced.^1^ For example, implementation in Sweden began in 2010 and since 2018 adults have had access to their online records.^2 3^ In Norway, implementation started in 2015 expanding to patients in three out of four regions by 2019.^4^ Since 2021 in the USA, the federally enacted 21st Century Cures Act mandated that providers offer all patients access to download their electronic health records without charge.^5^ Patients report many benefits from reading their clinical records including feeling more empowered, better understanding and remembering their treatment plan, and greater awareness about medications including possible adverse effects.^1 2 6–8^

While there is a growing interest in examining the risks of ORA with respect to patient privacy, this research agendum that largely focuses on safeguarding and proxy access^9–12^—for example, how patients might selectively hide information that they do not want visible on their online portals and decisions about when to ethically block access among adults or children in domestic abuse situations.^9 13–15^ A different privacy consideration is how ORA intersects with ‘surveillance capitalism’.^16^ This phrase, coined by the social scientist Zuboff refers to the monetisation of human experiences and behaviours on the internet.^17^ For example, personal data from internet searches may be sold to medical health insurance companies, direct to consumer drug advertisers or other for-profit enterprises.

An overlooked, unintended consequence of ORA is that detailed medical internet searches, social media use and the uptake of a new generation of chatbots powered by large language models (LLMs), exemplified by OpenAI’s GPT-4, Google’s Bard and Microsoft’s Bing AI, might expose patient data to new privacy risks. While these kinds of privacy risks do not constitute a robust reason to deny ORA (and very strong additional argumentation would be required to justify such a stance, taking us beyond the scope of the present paper), nonetheless, the practical realities of everyday internet use, patient accessible records and privacy regulations deserve scrutiny. This paper attempts to initiate that task. The paper surveys the risks associated with patient internet use, before examining the specific privacy risks associated with clinician documentation practices in the era of ORA. It concludes with a variety of multilevel suggestions for how privacy risks might be better mitigated.

## Potential privacy risks of patients using open notes

During clinic visits, patients misremember around half of what is communicated,^18^ and often fail to understand the health information conveyed by doctors.^19^ It is hypothesised that access to electronic health records may present a workaround solving a variety of face-to-face communication breakdowns that can arise in face-to-face visits.^20 21^ The suggestion is patients are afforded opportunities to pore over their records, and to remember, and better grasp what was conveyed by clinicians. While this seems like a sound supposition, it also seems reasonable to suggest access to the records alone may not facilitate improved understanding about what is documented—for at least two reasons.

First, while patients have experiential knowledge about their condition and health, most do not have in-depth medical expertise or a command of medical vernacular, even though some do become experts on their condition(s).^22 23^ Clinicians as domain experts, have a tendency to overestimate patients’ knowledge of specialist or technical language, failing to calibrate it effectively to lay levels of understanding^24^—a cluster of problems collectively referred to as ‘the curse of expertise’.^25^ This epistemic imbalance suggests it is unlikely that patients are fully equipped to understand all the detail housed in their electronic records.

Second, and relatedly, clinical records traditionally served as an aide memoire for clinicians, or as a tool for communicating detailed medical information to other providers, and not as a way to convey accessible health information to patients.^26^ Although significant proportions of surveyed physicians report changing how they write clinical information in the era of ORA including changing language perceived as critical of the patient, and modifying how they document sensitive information,^27–29^ there is evidence that the medical terminology embedded in notes is largely preserved. For example, in a US survey led by DesRoches of clinicians’ experiences with writing open notes, of 1628 clinicians (response rate 27%), 76% (n=966) reported that open notes did not affect the value of their notes for other clinicians.^29^ Furthermore, beyond the narrative reports written by clinicians, lab and test results, and medications, can be logged without any corresponding, clarificatory notes offered to patients.

Still, patients do report multiple benefits from ORA including (as noted above) feeling more empowered and better understanding their treatment plans and medications.^1 2 6–8^ This intimates that increased understanding might not derive from reading the records in isolation, but arises in conjunction with supplementary resources. Preliminary findings strongly indicate this could indeed be the case. For example, in the largest patient survey of open notes conducted in the USA at three health centres, of 29 656 adult patients (response rate: 22%), 23 576 (79%) reported reading at least 1 note, and among them, 19 411 (82%) reported they were taking or had been prescribed a medication in the past 12 months; of them, 32% said access made them ‘seek more information’ about their medications.^8^ Furthermore, among patients with serious mental health diagnoses, the proportion was higher: 39% reported seeking more information after reading their clinicians’ notes.^30^

### Prevalence of internet use for health information searches

This constellation of factors invites nontrivial questions about where and how patients might supplement deficits in their understanding. Notably, it is now well established that consumers use the internet to seek health information^31^ with an estimated 7% of Google searches health related.^32^ Recent findings show, more four in ten Americans use Google instead of seeing a doctor,^33^ and around half of Europeans search for health information online.^34^ When it comes to social media, the statistics are even more arresting with an estimated 4.8 billion social media users globally, comprising 6 in 10 of the world’s population.^35^ Exchanging and accessing health information is a common reason for using social media^36^; for example, in in one recent US survey, three in four respondents said they relied on Facebook and Twitter for COVID-19 health information.^37^ In a study in Germany, Braun *et al* found that the internet was the most important source of information for patients with cancer (75%, n=308), with half the respondents (49.2%, n=196) reporting that a cancer diagnosis was the stimulus for starting to engage with social media.^38^

To reiterate, although the connection between ORA and patient internet use has not been directly explored by health services researchers, since patients frequently use online resources to strengthen knowledge about their health, it is not a leap to suppose many may be inputting sensitive information into websites to augment their understanding. Although they may already be doing so regardless of access to their electronic health records, with online access such searches are likely to be more detailed: patients may use the internet to decipher detailed test results, to translate the technical language embedded in documentation and/or to avoid confusions.

### Risks from search engines and social media

This usage invites questions about the extent of privacy exposures arising when patients use search engines or social media, and privacy risks differ between countries and regions. In the USA, the 1996 Health Insurance Portability and Accountability Act (HIPAA) created national standards in the USA to protect patients’ health information from being shared by ‘covered entities’—that is providers—to other third parties. In the epoch of the internet in the USA, HIPAA lacks teeth to protect patient privacy from surveillance capitalism. While generic symptom searches risk exposing personal consumer information to tech giants, using search engines or social media as vehicles for more precise understanding about the information housed in their records—perhaps ‘cutting and pasting’ detailed information into browsers or on social media—could lead to more meaningful exposures. Furthermore, in the USA, the 21st century Cures Act mandates that patient’s ORA is also available in a downloadable form for use with other apps.^39^

In the European Union, under the General Data Protection Regulation (GDPR), citizens have greater control over their personal information.^40^ Without informed consent, or unless exceptional circumstances are met, such as public health justifications, companies are prohibited from processing and trading in, ‘data concerning health’ defined as ‘personal data related to the physical or mental health of a natural person, including the provision of healthcare services, which reveal information about his or her health status.’^40^

Against the foregoing considerations, if patients are using search engines, social media, or other smartphone applications (apps) to strengthen understanding about their care, do any such privacy exposures constitute an ethical problem or one that constitutes a privacy violation? On the face of it, it might seem that the individual responsibility is with the actor who chooses to use the internet to supplement their understanding of their medical information—that any related exposures do not constitute privacy violations and instead constitute freely made decisions, exercised without external pressures.

This, however, oversimplifies the sociotechnical context of internet use, exaggerating the freedom that consumers have when they turn to online health resources. One reason for scepticism is that consent processes do not meet threshold elements or preconditions for consent in terms of competence (of consumers’ capacity to understand and decide), and of voluntariness (in making decisions), and of requisite information elements (the disclosure of salient information relevant to consent).^41^

Consider the first of these, competence to decide. Even in the EU context where data harvesting is more stringently regulated, when offered the option to give consent to data collection via apps, consumers are also obliged to read through and understand labyrinthine terms and conditions. The reality is these are often unfeasibly onerous. In 2008, it was estimated the average internet user would need 76 days per year to wade through every internet privacy policy before deliberating over consenting, a figure undoubtedly higher today.^42^ Zuboff argues that the very minutiae and scale of the small print is one reason consumers are inclined to put their privacy at risk.^17^ Simply put, the standard of competence required to meaningfully consent is often unreasonable.

When it comes to voluntariness—of consumers making decisions without external pressures—there are also reasons for doubt. The internet is now a domestic necessity akin to running water, electricity, or plumbing. Most internet users appear cognizant that their personal data might be monetised or sold to third parties. In 2018, a survey by Rock Health reported that only 1 in 10 Americans were willing to share their health data with tech giants such as Facebook and Amazon.^43^ Recent Pew Research studies show most Americans distrust private companies with an estimated 8 in 10 believing their personal data is less secure now, and that it is not possible to go through daily life without being tracked.^44^ Despite these reservations, as we have seen, health-related internet searches and social media use are commonplace. As Zuboff argues, the explanation for this apparent tension between risking data exposure and using online resources, is that the choice to opt out is essentially an ersatz one: the internet is so essential for social participation that it, ‘produces a psychic numbing that inures us to the realities of being tracked, parsed, mined and codified’.^17^

A related reason to doubt that threshold conditions of voluntariness in consent processes are met comes from the increasing pressures on health systems and their staff. Patients prefer to receive health information directly from their doctor; for example, in the study by Braun *et al*, 85% said they wanted to get information from their doctors (n=342), yet the internet was reportedly the most important source of information for patients with cancer (75%, n=308).^38^ Physician time is now one of the scarcest resources in medicine. The COVID-19 pandemic oversaw a flight of physicians from primary care^45^; and globally, the WHO predicts a worldwide shortage of 10 million healthcare workers by 2030.^46^ In England, for example, as of April 2023, nearly 5 million patients waited more than 2 weeks to obtain a general practitioner (GP) appointment.^47^ Even prior to the pandemic, health systems have increasingly been strained with ageing populations, and with more people suffering chronic illnesses for longer. Perhaps unsurprisingly, surveys in the UK and the USA show that physician burnout is at an all-time high.^48 49^ Combined, studies show people are often acutely aware of the pressures on doctors, and of the rationing involved in health systems,^50^ with many reporting using internet resources to avoid asking too many questions in clinic visits, out of fear of taking up too much time in visits, or to avoid ‘doctor-bothering’.^51 52^

A third reason to doubt consent processes are being met comes from analyses of informational elements in health apps.^53^ For example, a recent analysis of privacy policies of 36 top-ranked apps for smoking cessation and depression, available for download in popular app stores, found that 29 transmitted data to services provided by Facebook or Google, yet only 12 accurately disclosed this in a privacy policy.^54^

### Risks from LLM-powered chatbots

Aside from the range of elevated exposures via search engines and social media, the discussed risks to patient privacy are likely to be even graver with LLMs-powered chatbots, such as ChatGPT. This new generation of chatbots offers an unprecedented level of conversational fluency in computer-human interactions.^55^ Unlike traditional search engines which offer lists of webpages, LLM-based chatbots facilitate exchanges that mimic dialogue and ‘remember’ previous prompts, helping to create the perception of smoother exchanges. LLMs use massive amounts of past data to predict the next word in a sequence. This probabilistic process combined with other technical advances means these models are well suited to recognising, summarising and generating content.^56^ However, it is important to emphasise that these chatbots are not without significant limitations: the quality of the data on which they are trained means they may generate discriminatory advice (so-called ‘algorithmic biases’) that could worsen racial, ageist or gender discrimination in care; they are prone to making things up (‘hallucinations’); and could offer harmful medical advice.^57^

The extent to which patients use generative-AI chatbots to augment understanding about their healthcare is only beginning to be explored. In June 2023, in a survey conducted by Medical Economics, 8 in 10 Americans surveyed believed these tools had the potential to improve the quality of healthcare, reduce costs and increase the accessibility of care.^58^ Precisely because these chatbots are user-friendly, however, consumers may be seduced into anthropomorphising the interactions. For example, for low risk health complaints, one experimental study demonstrated ChatGPT responses to patients’ queries were only weakly distinguishable from clinician responses with patients trusting chatbot responses.^59^ These attributes could render LLMs such as Chat-GPT particularly potent extractors of sensitive and detailed health information.^55 60^

## Potential privacy risks of clinicians writing open notes

Potential privacy exposures do not only arise from the patient side: using LLM-powered chatbots clinicians may inadvertently expose sensitive patient data too. For example, in July 2023, five hospitals in Australia’s South Metropolitan Health Service, in Perth, were instructed to stop using ChatGPT after it was discovered some staff had been using the chatbot to write clinical notes.^61^ In the USA, in a June 2023 Medical Economics study, more than 1 in 10 surveyed healthcare professionals reported adopting generative AI chatbots such as ChatGPT, and nearly 50% expressed an intent to use these technologies in the future for tasks such as data entry, medical scheduling or research.^58^

Despite the (aforementioned) limitations associated with these tools, their greatest current promise lies in assisting clinicians with administrative tasks. Here, the potential should not be underemphasised.^56^ LLM-powered chatbots have the capacity to summarise material in a requested style or tone suitable for a wide range of lay readers even removing jargon or phrasing that may interfere with medical understanding, by couching responses at different literacy levels, or adopting an empathic tone.^62^

Moreover, given the pressures on them, clinician motivations for clinicians adopting LLMs such as Chat-GPT are clear, and as we’ve seen, preliminary evidence suggests that at least some doctors are turning to generative AI to assist with workplace demands. Furthermore, ORA may invite new work burdens of its own since the electronic health record now serves as a communication tool, not just for other providers but also for patients. A key trend in physician surveys is widespread fear that these measures, and subsequent patient contact as a result of ORA, will increase in workloads.^27 63–65^ Evidence suggests that clinicians recognise adjustments to documentation may be needed so that information is recorded in understandable and sensitive ways.^9 27 63^ Following implementation of open notes, in the survey by DesRoches, 36% (n=463) of clinicians reported spending longer writing notes, with 58% (n=422) changing language that might be perceived as critical of patients and 49% (n=372) changing how they documented sensitive clinical, mental health or social information.^29^ While there is a paucity of objective evidence to demonstrate patient online access does increase work burdens, some findings do suggest an increase in clerical workload with patient email traffic increasing, in particular, following the release of test results.^66–68^ At least some of this increased contact, it seems reasonable to surmise, may be driven by patients who are confused or desire clarifications about their medical information.

Again, however, when clinicians adopt these chatbots to assist with documentation they risk exposing sensitive patient information. For these reasons, in June 2023, the American Psychiatric Association issued guidance strongly opposing physicians entering patient data into generative AI systems.^69^ And in July 2023, responding to reports of physicians using ChatGPT to write medical notes, the Australian Medical Association, called for stronger AI regulations.^70^

## Suggestions to reduce privacy risks of open notes

Patients, and clinicians, may be concerned about the potential privacy exposures associated with sharing clinical notes and internet use, and to mitigate risks, several suggestions could be considered.

First, there are serious deficits in health and social care professionals’ digital literacy, including with respect to the ethical implications of digital tools.^71–73^ Similarly, patients may also need training to become more knowledgeable about how to use digital innovations in healthcare including the positive and negative aspects of these tools.^74^ Brief educational interventions could help clinicians and patients become more aware about the risks associated with surveillance capitalism, including of using internet search engines, social media and generative AI chatbots, to supplement understanding of information housed in electronic medical records, or—in the case of clinicians—to assist with writing documentation patients might read. Increasingly, health organisations are becoming aware of the need to offer guidance to patients about the benefits and risks of accessing ORA, for example, via leaflets and practice websites^9 11 12^; and guidance about privacy risks could also be discussed. Similarly, in undergraduate medical education, GP training programmes, and continuing professional development courses, brief training about the internet and privacy could improve clinicians’ understanding about why innovations such as ChatGPT pose risks to patient data.^75^

Second, health systems could strive to improve the understandability of health information curated in patient accessible records. This could be achieved via improved patient codesign of portals, including embedding and curating access to patient-friendly resources that are linked to, and housed securely within, the electronic records.^20^ As noted, patients also prefer to receive information from their physicians. Therefore, to reduce the risks of patients actively supplementing their understanding with internet or chatbot searches beyond the portal, it would be valuable to augment the quality of the narrative reports written by clinicians. To that end, given that LLM-powered chatbots may offer significant promise in assisting with writing medical notes, health systems could seek to embed such tools within electronic record systems only if these tools comply with patient privacy laws. Already there are promising efforts in this direction. For example, in the USA, an Azure HIPAA compliant GPT-4 service now exists,^76^ and Epic—which has the largest share of the hospital medical record market^77^—is piloting the integration of HIPAA-compliant GPT services.^78^ Given the limitations and challenges associated with LLM-powered chatbots, including the tendency to ‘hallucinate’, the potential for bias, and inconsistencies in summative responses, clinicians may need guidance about the careful adoption and use of these tools and should be strongly advised that such tools might be valuable in assisting with writing documentation but not replace clinicians in undertaking this task.

Third, at a civic level, questions about data ownership and consent for using health data need to be resolved.^79^ It is worth emphasising that, even when it comes to the USA’s HIPAA-compliant LLM-powered chatbots, establishing whether patients consent to use their electronic record as training data, is worthy of further scrutiny. Indeed, beyond health data, legal challenges relating to copyright infringements, by writers and artists,^80^ have already been raised on the grounds that LLM models scrape public data from the internet to train their models.^81^ Authorities in the EU are currently reviewing whether, without obtaining informed consent, OpenAI’s ChatGPT complies with GDPR regulations and meets the requirement of public health justifications.^82^

## Conclusions

ORA affords patients many benefits including feeling more in control of, and knowledgeable about, their healthcare. However, currently, these benefits are unlikely to accrue without supplementation from internet-based resources. ORA access combined with the intrinsicality of internet use in daily life, surveillance capitalism, the pressures on health systems, and the challenges of readily availing of health services, create the perfect storm for privacy exposures. It is imperative for patients and clinicians to become more aware of these privacy risks. Health systems should also, as a matter of exigency, outline policies that uphold privacy in the use of LLM chatbots to assist clinicians with documentation. While debates about regulating the power of tech giants continue to rage at political and legislative levels, the elevated risks of exposing private health information with online record access should also be considered.

## Data Availability

No data are available.
